# Immunomodulatory effects of *trans*-anethole-treated *Staphylococcus aureus* Newman strain

**DOI:** 10.1038/s41598-023-34138-3

**Published:** 2023-04-27

**Authors:** Paweł Kwiatkowski, Karolina Rogulska, Agata Pruss, Monika Sienkiewicz, Barbara Dołęgowska, Iwona Wojciechowska-Koszko

**Affiliations:** 1 Department of Diagnostic Immunology, Pomeranian Medical University in Szczecin, 72 Powstancow Wielkopolskich, 70-111 Szczecin, Poland; 2 Department of Laboratory Medicine, Pomeranian Medical University in Szczecin, 72 Powstancow Wielkopolskich, 70-111 Szczecin, Poland; 3 Department of Pharmaceutical Microbiology and Microbiological Diagnostic, Medical University of Lodz, Muszynskiego St. 1, 90-151 Lodz, Poland

**Keywords:** Plant sciences, Infection

## Abstract

In our former studies based on a human whole-blood model infected with *trans*-anethole (TA)-treated *Staphylococcus aureus* Newman strain, we have observed that selected parameters/mechanisms of innate and acquired immune response were more enhanced in comparison to samples infected with non-treated bacteria. Due to this observation, the current study aimed to evaluate the concentration of selected proteins involved in both types of responses (IL-1α, IL-1β, IL-2, IL-6, IL-12, IL-17, TNF-α, IFN-γ, G-CSF, C5a, CCL1—CCL5, CXCL1, CXCL2, CXCL9—CXCL11, MMP-8, TLR2, and PGLYRP1) in healthy participants' plasma after blood stimulation of TA-treated *S. aureus* Newman strain. Determination of analyzed protein concentration was conducted using Luminex and ELISA assays. Based on the results, it has been proven that the immunomodulatory potential of TA-treated *S. aureus* Newman strain on increasing IL-1β, IL-6, TNF-α, IL-12, G-CSF, C5a, CCL2—CCL4, CXCL1, CXCL2, MMP-8 and PGLYRP1 levels in plasma. Moreover, it has been also demonstrated an association between TNF-α and CCL4 in a blood model infected with TA-treated cells. More research is warranted to find more underlying mechanisms involved in the effects of TA-treated *S. aureus* Newman in human blood, mainly whether the observed “immunity boost” can be regulated after bacteria elimination. Therefore, the potential of TA should be further explored to understand under which conditions it might help treat or prevent infections caused by *S. aureus*.

## Introduction

The presence of *Staphylococcus aureus* in the blood results in the rapid activation of an immune response, primarily an innate immune response. Because neutrophils are the most abundant leukocytes, it is considered that these cells (through phagocytosis, production of reactive oxygen species, formation of extracellular traps [NETs or NETosis], and degranulation) are mainly involved in the immune processes that lead to the destruction of these bacteria^1^. Neutrophils are equipped with several pattern/pathogen recognition receptors (PRRs), including, e.g., Toll-like (TLRs), nucleotide-binding oligomerization domain-like, C-type lectin, or peptidoglycan (PG) recognition (PGRPs) receptors which recognize pathogen-associated molecular patterns (PAMPs). For instance, the main PAMP of *S. aureus* is considered PG, which is recognized, e.g., through TLR2 and PGRP-S (also known as PGLYRP1 or Tag7), leading to their activation^2,3^. Interestingly, PG is also capable of inducing matrix metalloproteinases (MMPs) and affects the elevated concentration of these enzymes (derived from neutrophils) in the blood^4^.

As a consequence, the activation of neutrophils leads to produce pro-inflammatory cytokines (e.g., tumor necrosis factor [TNF] α—TNF-α, interleukin [IL] 1α—IL-1α, IL-1β, IL-6, IL-12, IL-18), chemokines (e.g., [C–C motif ligand] 2—CCL2, CCL3, CCL4, [C-X-C motif ligand] 1—CXCL1, CXCL2, CXCL8—[IL-8], CXCL9, CXCL10, CXCL11), granulocyte-colony stimulating factor (G-CSF), and other proteins including, e.g., angiogenic and fibrogenic factors)^5,6^. It is worth mentioning that neutrophil activation can be modulated by cytokines secreted by other immune cells, including, e.g., NK cells, circulating dendritic cells (DCs), monocytes, eosinophils, basophils or even acquired immune cells (γδ T, Th1, Th2, and Th17). In response to this, numerous proteins appear in the blood, including i.a. TNF-α, interferon (IFN)-γ, IL-1β, IL-2, IL-4, IL-6, IL-10, IL-12, IL-17, complement component 5a (C5a), or transforming growth factor (TGF-β)^7–10^. Hence, the mechanism of cytokine secretion by immune cells forms a complex and integrated network whose task is to shape the immune response.

Recently, there has been a growing interest in substances of natural origin, including essential oils (EOs) and their main compounds (EOCs). They have been proven to have numerous biological properties, including immunomodulatory^11,12^. Notably, most articles deal with the inhibitory effects of EOs/EOCs on cytokine and chemokine production. In addition, there is a growing number of studies in which the authors test the immunomodulatory properties of EOs/EOCs after stimulation of immunocompetent cells with whole bacteria or their components, such as lipopolysaccharide (LPS)^12^. For example, a study performed by Martins et al*.*^13^ showed that BALB/c mice infected with *S. aureus* ATCC 33,591 and then stimulated with citral EO reduced levels of IL-1β, IL-6, and TNF-α, compared to non-stimulated mice. Furthermore, a study by Zonfrillo et al*.*^14^ showed that human monocyte-derived macrophages stimulated with LPS followed by eucalyptus EO or eucalyptol produced lower IL-6, TNF-α, CCL2, CCL5, CXCL8, CXCL9, and CXCL10 levels compared to cells stimulated only with LPS.

It is worth noting that the studies mentioned above involve the stimulation of immune cells with antigens that have not been previously pre-treated with the active substance. As Nazzaro et al*.*^15^ point out, EOs and EOCs show activity against various bacterial targets, including LPS or PG, leading to a complete morphological change in cells. For instance, Yu et al*.*^16^ concluded that the *trans*-anethole (TA, a natural compound found in many EOs, including fennel, anise, or star anise) mechanism of action against bacteria might be associated with cell wall degradation. Interestingly, our former studies proved that the *S. aureus* Newman strain cultured on a medium containing a subinhibitory concentration of TA was characterized by structural changes in the cell membrane and PG^17^. Since PG in *S. aureus* is considered one of the significant PAMP, it is worth asking here whether changes in its structure after TA stimulation can enhance or suppress the activation of immune cells. In a way, we have already taken some steps in this direction and noticed increased activation of the immune system^17,18^. One has proven that TA-treated *S. aureus* Newman added to human blood increased phagocytosis efficiency, intracellular killing, and CXCL8 production^17^. Moreover, TA-treated bacteria had lower staphyloxanthin levels and were characterized by reduced antioxidant activity compared to untreated bacteria. In another study, it has been found that lymphocytes isolated from TA-treated *S. aureus* Newman-infected blood showed significantly higher expression of *IL1B*, *IL6*, *IL10*, *TNF*, and *TLR2* compared to blood samples infected with non-TA-treated bacteria^18^.

Our previous studies proposed a model of human blood infected with TA-treated *S. aureus* Newman, which contributed to the study of selected components/mechanisms of the innate and acquired immune response^17,18^. This time, we want to analyze the abundance of selected proteins secreted into the blood during the immune response against the TA-treated *S. aureus* Newman strain. We realize that the molecules' production during the inflammatory process forms a complex network. However, the results obtained in this study will serve as a starting point for further research into understanding the effects of TA-treated *S. aureus* on immune system activation. Therefore, the present study is aimed to evaluate the concentration of selected cytokines (IL-1α, IL-1β, IL-2, IL-6, IL-12, IL-17, TNF-α, IFN-γ, G-CSF) chemokines (C5a, CCL1, CCL2, CCL3, CCL4, CCL5, CXCL1, CXCL2, CXCL9, CXCL10, CXCL11), enzymes (MMP-8), and PRRs (TLR2, PGLYRP1) in healthy participants (recruited based on medical history and average complete blood count—CBC) plasma after blood stimulation of TA-treated *S. aureus* Newman strain. Additional attention was paid to the correlation of protein abundance among human whole-blood models infected with TA-treated *S. aureus* Newman strain and non-treated bacteria (control).

## Materials and methods

### Strain and culture condition

The study used *S. aureus* ATCC 25904 (Newman) reference strain. Before each experiment step, the strain was cultured in aerobic conditions at 37 °C for 18 h on Columbia agar supplemented with 5% sheep blood (bioMérieux, Warsaw, Poland).

### Participant recruitment and ethical approval

The study was performed between October 2021 and November 2021 at the Immunology Laboratory, Independent Public Clinical Hospital No. 2 of the Pomeranian Medical University in Szczecin, Poland. A total of 20 healthy participants (age = 21–64 [median age = 43]; sex ratio = 2:18 [10% male]; Caucasian ethnic group) were examined. Detailed characteristics of participants by age and sex are presented in Supplementary Materials—Table S1. All participants were interviewed on their general health status, including their medical history (e.g., no exposure to *S. aureus* and other infections requiring antibiotic therapy and no hospitalization history for the past 6 months), which determined their eligibility for the study. Additionally, each qualified participant's body temperature was measured at the axilla using the digital thermometer, which was expected.

The study was approved by the Ethical Committee of the Pomeranian Medical University in Szczecin (approval number: KB-0012/200/2020) and was conducted following the Declaration of Helsinki. Each participant qualified for the study was thoroughly informed about the purpose and nature of the study. In addition, each participant gave informed consent to participate in the study. Before analysis, participant data and information were anonymized and disidentified.

### A human whole-blood model infected with *S. aureus* Newman

Two venous blood samples (∼ 2.5 ml and ∼ 7.5 ml) were taken from each participant by a qualified medical person and collected in S-Monovette tubes (Sarstedt, Nümbrecht, Germany) containing ethylenediaminetetraacetic acid. The first tube (∼ 2.5 ml) was used for the complete blood count (CBC) evaluation using the Sysmex XN-2000 (Sysmex Europe, GmbH, Norderstedt, Germany) in Department of Laboratory Diagnostics, Public Clinical Hospital No. 2 in Szczecin, Poland. The remaining blood sample (∼ 7.5 ml) was used to design a model infected with staphylococcal cells according to former studies^17^ using reagents purchased from Merck KGaA (Poznan, Poland). Briefly, *S. aureus* Newman was cultured on Columbia agar supplemented with 5% sheep blood (bioMérieux, Warsaw, Poland) and incubated in aerobic conditions for 18 h at 37 °C. Then, the grown bacteria colonies were seeded on the following media: A—control medium (Mueller–Hinton agar, MHA), B—MHA supplemented with 1% (v/v) Tween 80 (the substance increasing the solubility of TA in concentration not influence the growth of staphylococci), and C—MHA supplemented with 1% (v/v) Tween 80 and TA at the subinhibitory concentration (5%, v/v)—the concentration obtained in the previous study^18^. After an incubation period, colonies were washed five times using phosphate-buffered saline (PBS, pH 7.4, sterile-filtered) and adjusted to the turbidity of 4 McFarland scale. Participants’ blood (∼ 7.5 ml) was divided into four equal volumes. Three were infected with the prepared staphylococcal suspension (obtained from A–C media) to form A (control), B, and C infection models. The volume of bacterial suspension did not exceed 14.3% (v/v) of the total sample volume. Samples were mixed by inversion and incubated for 2 h at 37 °C with gentle rotation. Concurrently, the non-infected whole human blood model (N model) was performed using PBS at the same condition mentioned above.

**Figure 1:**
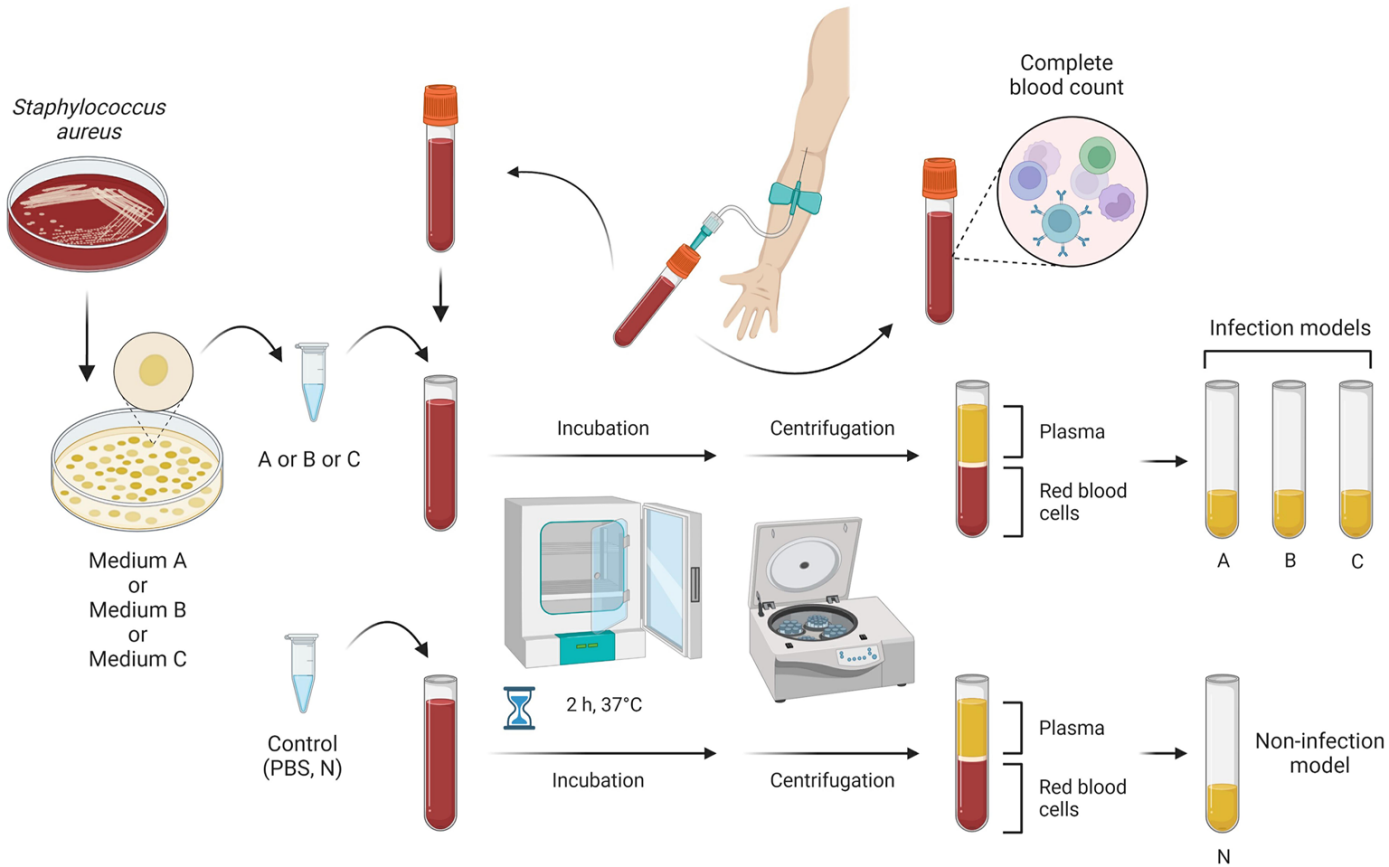
Schematic diagram of the experiment. Created with BioRender.com.

After completing the studies, the blood samples were immediately centrifuged (524 × g, 10 min, 20 °C), and the plasma was transferred to another tube and frozen at − 80 °C until assayed. A schematic diagram of the experimental setup is graphically presented in Fig. 1.

### Luminex assay

IL-1α [IL-1F1], IL-1β [IL-1F2], IL-2, IL-6, IL-12 [IL-23 p40], IL-17 [IL-17A], TNF-α, IFN-γ, G-CSF, C5a, CCL1 [I-309, TCA-3], CCL2 [JE, MCP-1], CCL3 [MIP-1α], CCL4 [MIP-1β], CCL5 [RANTES], CXCL1 [GROα, KC, CINC-1], CXCL2 [GROβ, MIP-2, CINC-3], CXCL9 [MIG], CXCL10 [IP-10, CRG-2], CXCL11 [I-TAC], and MMP-8 concentrations were measured in plasma samples (obtained from N–C models) by multiplex fluorescent bead-based immunoassays (Luminex Corporation, Austin, TX, United States) using commercial R&D Systems Luminex Discovery Assay Human Premixed Multi-Analyte Kits (R&D Systems, Minneapolis, MN, USA). All steps were performed according to the manufacturer's protocol. All plates were read and analyzed on a Luminex 200 analyzer (Luminex Corporation, Austin, TX, USA), and analyte concentrations were determined from six standard curves showing mean fluorescence intensity (MFI) vs. protein concentration. The study was carried out in duplicate.

### Enzyme-linked immunosorbent (ELISA) assay

To quantify selected PRRs concentration in plasma obtained from N–C models, TLR2 (EH459RB) and PGLYRP1 (EHPGLYRP1) Human ELISA™ Kits (Invitrogen, Waltham, MA, USA) were used, respectively. All steps were performed according to the manufacturer's protocol. PRRs concentration was determined from seven standard curves showing absorbances vs. protein concentration. The study was carried out in duplicate.

### Statistical analysis

The Shapiro test of normality was run on the outcome variables, and following this, a statistical significance between groups was measured using Kruskal–Wallis one-way ANOVA with Dunn's multiple comparison test. Parametric correlations were made according to Pearson's correlation. Results *P* < 0.05 were considered statistically significant. All statistical analyses were performed using GraphPad Prism 8.0.1 (GraphPad Software Inc., San Diego, CA, USA).

## Results

### Evaluation of CBC parameters of participants

**Figure 2:**
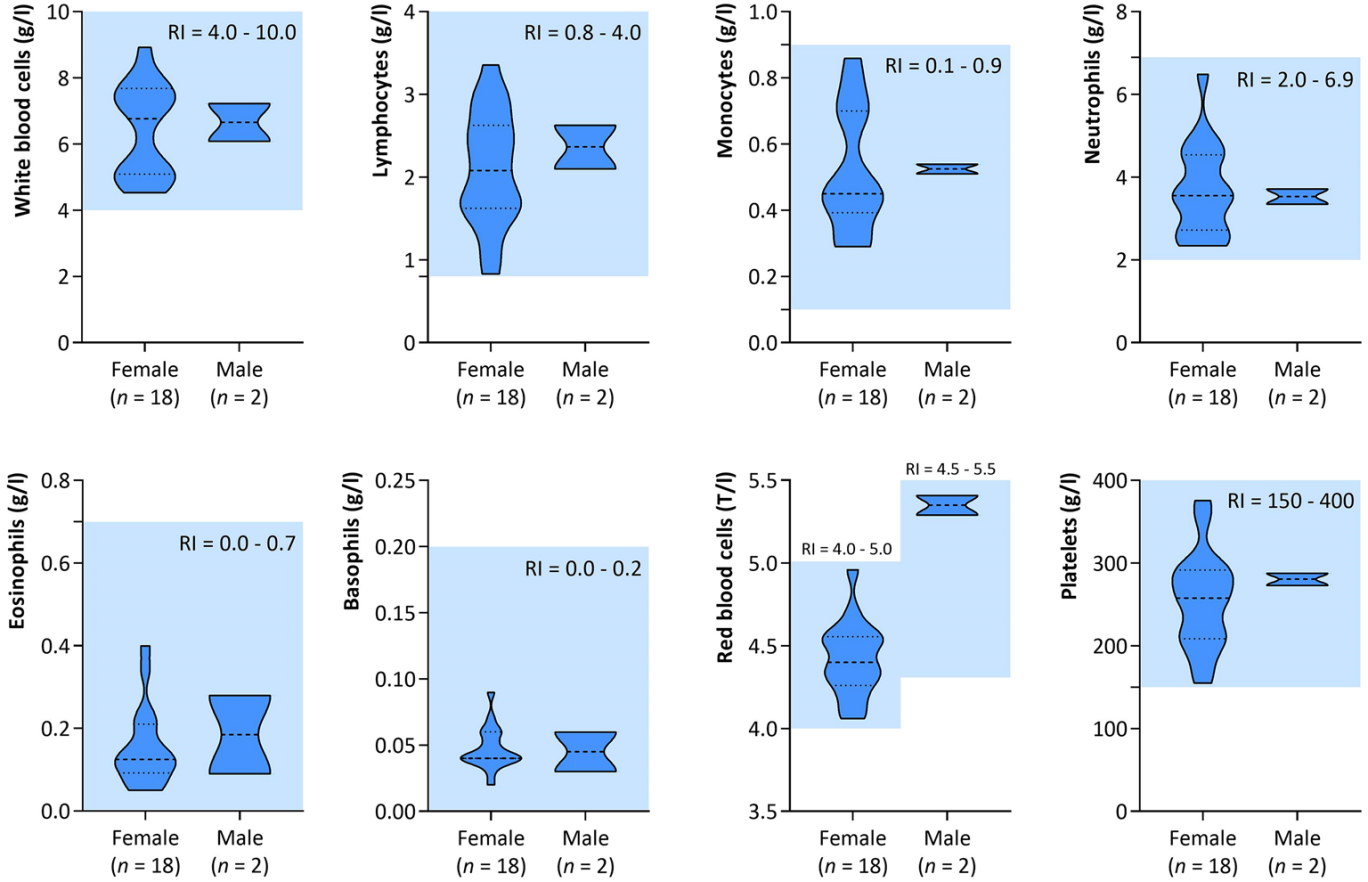
Selected hematological parameters of blood samples isolated from healthy participants (*n* = 20). The light blue color mark—the reference interval (RI) for each hematological parameter. Violin plots illustrate the data distribution, median and interquartile ranges.

It was revealed that selected hematological parameters (the level of white blood cells, lymphocytes, neutrophils, monocytes, eosinophils, basophils, red blood cells, and platelets) of the blood samples of twentieth participants were in the range of laboratory-specific reference intervals (Fig. 2). Detailed characteristic of hematological parameters is presented in Supplementary Materials—Table S1.

### Determination of cytokine and chemokine concentration

**Figure 3:**
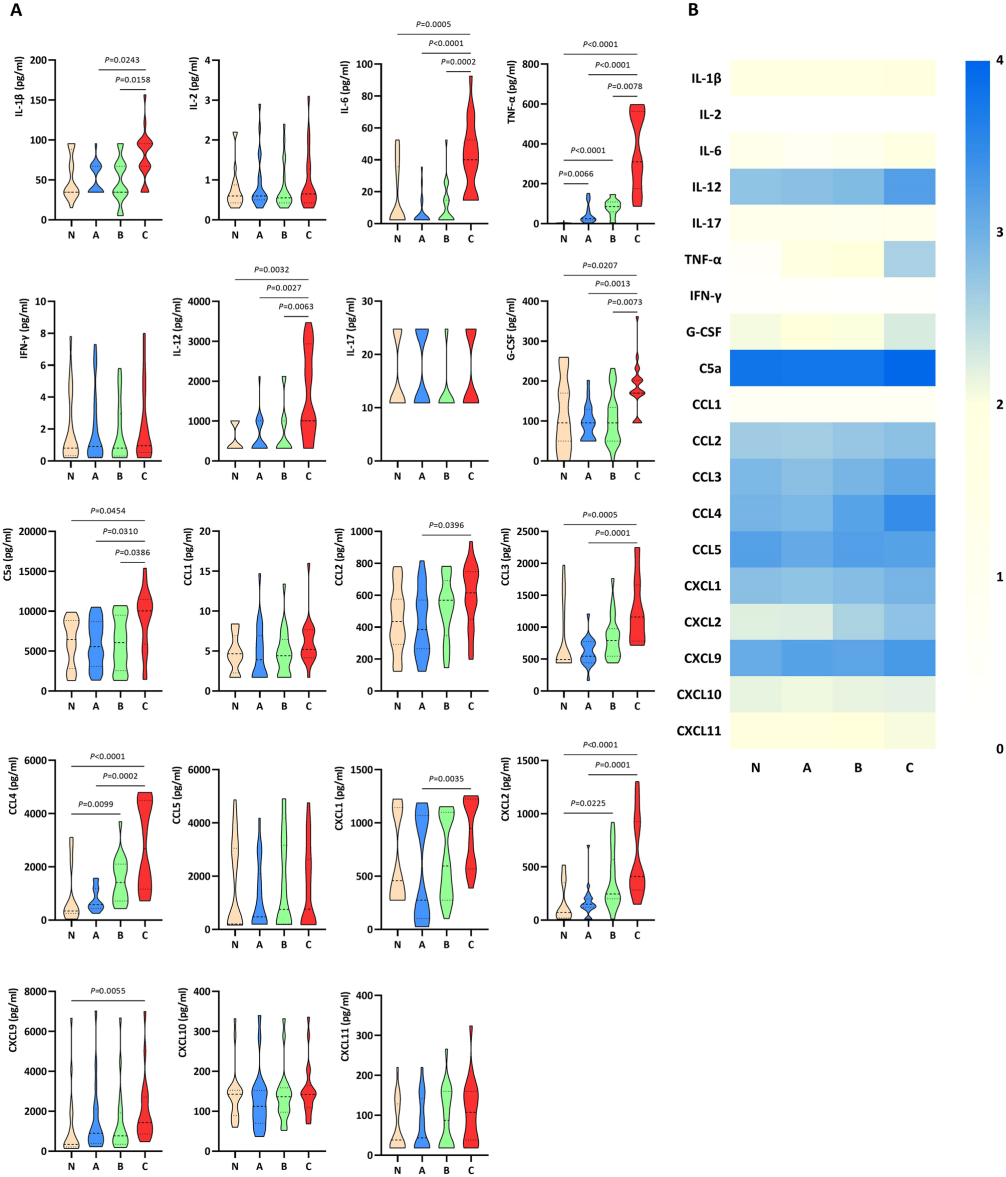
Violin plots (**A**) and heatmap (**B**) showing the mean cytokine and chemokine concentrations in the plasma isolated from whole human blood samples (*n* = 20) non-infected (N model) and infected with the *Staphylococcus aureus* Newman strain precultured on Mueller–Hinton agar: non-supplemented (A model—control); supplemented with 1% (v/v) Tween 80 (B model); supplemented with 1% (v/v) Tween 80 and *trans*-anethole at the subinhibitory concentration (5%, v/v, C model). Violin plots illustrate the data distribution, median and interquartile ranges. The heatmap represents a mean cytokine/chemokine log concentration (+ 4 blue to 0 light yellow).

Figure 3 presents the results concerning the concentration of cytokines and chemokines in the plasma isolated from human whole-blood samples non-infected (N model) and infected with the *S. aureus* Newman strain precultured on A-C media (A-C models). Furthermore, the detailed statistical parameters of results and *P* values of the N–C models’ data obtained in the experiment are listed in Supplementary Materials—Tables S2, S3.

All cytokines in plasma were detected based on the tests performed, except IL-1α, the mean values of which were below the thresholds of detection. IL-12 was the most abundant cytokine expressed with a mean of 559.5 pg/ml in the N model, 615.2 pg/ml in the A model (control), and 726.6 pg/ml in the B and 1647.1 pg/ml in the C model. On the contrary, IL-2, IFN-γ, and IL-17 were very weakly expressed in all models (N–C) with a mean of 0.9 pg/ml, 1.9 pg/ml, and 15.6 pg/ml, respectively.

Considering all the cytokines analyzed, only TNF-α had statistically higher concentrations in the infected models (A–C; mean value = 166.0 pg/ml) compared to the non-infected model (N; mean value = 4.0 pg/ml) (*P* ≤ 0.0066). Mean IL-1β, IL-6, IL-12, TNF-α, and G-CSF concentrations were higher in the C model compared to the A model and B model with significant *P* values ≤ 0.0243 and ≤ 0.0158, respectively. It was most noticed in the case of TNF-α, where an approx. eightfold higher concentration of this cytokine was observed in the C model (mean value: 367.6 pg/ml) compared to the A model (control; 48.3 pg/ml). Furthermore, several cytokines (IL-6, IL-12, TNF-α, G-CSF) showed statistically higher concentrations in the C model in comparison to the N model (*P* ≤ 0.0207), excluding IL-1β, the level of which were higher, but these differences were not statistically significant. Additionally, there were no statistically significant differences in IL-2, IL-17, and IFN-γ levels at analyzed N–C models.

All chemokines in plasma were detected based on the tests conducted. C5a was the most abundant chemokine in N–C models, and its mean concentration ranged from 5911.8 to 9014.3 pg/ml. Among all chemokines, only CCL1 was very weakly expressed in N–C models with a mean of 5.0 pg/ml. Mean C5a, CCL2, CCL3, CCL4, CXCL1, and CXCL2 concentrations were statistically more significant in the C model compared to the A model (control; *P* ≤ 0.0396). It is worth emphasizing that an almost 4 × increase in CCL4 concentration was observed in the C model (mean value: 2830.8 pg/ml) compared to the A model (772.2 pg/ml). Moreover, both B and C models had higher concentrations of CCL4 and CXCL2 than the N model, with significant *P* values ≤ 0.0225 and ≤ 0.0001, respectively. In addition, CCL3 and CXCL9 had statistically higher concentrations in the C model than in the N model (*P* ≤ 0.0055), with no statistically significant results among the remaining chemokines.

### Determination of MMP-8 and selected PRRs concentrations

**Figure 4:**
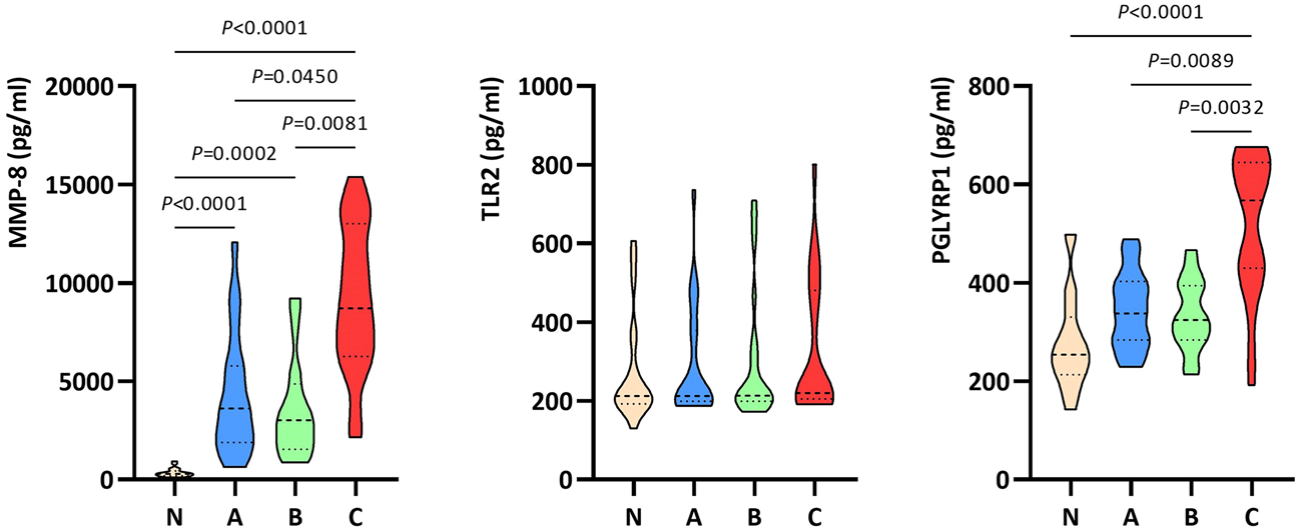
Violin plots showing the mean MMP-8, TLR2, and PGLYRP1 concentrations in the plasma isolated from whole human blood samples (*n* = 20) non-infected (N model) and infected with the *Staphylococcus aureus* Newman strain precultured on Mueller–Hinton agar: non-supplemented (A model—control); supplemented with 1% (v/v) Tween 80 (B model); supplemented with 1% (v/v) Tween 80 and *trans*-anethole at the subinhibitory concentration (5%, v/v, C model). Violin plots illustrate the data distribution, median and interquartile ranges.

Figure 4 presents the results concerning the mean MMP-8, TLR2, and PGLYRP1 concentrations in the plasma isolated from human whole-blood samples non-infected (N model) and infected with the *S. aureus* Newman strain precultured on A–C media (A–C models). Moreover, the detailed statistical parameters of results and *P* values of the N–C models' data obtained in the experiment are listed in Supplementary Materials—Tables S1, S2.

As can be seen in Fig. 4, only MMP-8 had statistically higher concentrations in the infected models (A–C; mean value = 5689.5 pg/ml) compared to the non-infected model (N; mean value = 349.4 pg/ml) (*P* ≤ 0.0002). Moreover, mean MMP-8 and PGLYRP1 concentrations were statistically higher in the C model compared to A (control) and B models with significant *P* values ≤ 0.0454 and ≤ 0.0386, respectively. In addition, TLR2 had a slightly higher mean concentration in the C model than in the A model, but no statistically significant results were observed.

### Correlation analysis of proteins in selected models

**Figure 5:**
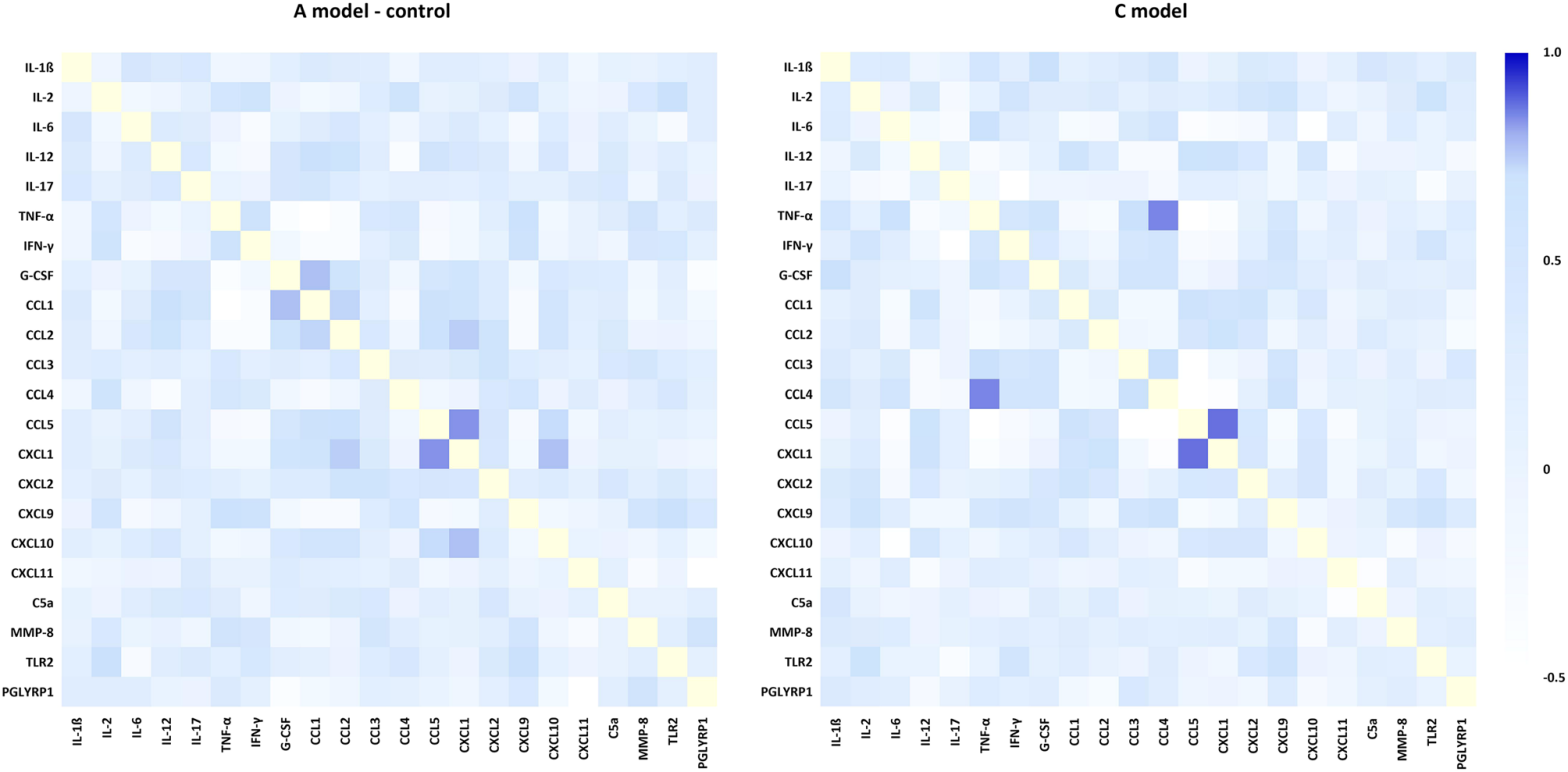
Pearson's correlation analysis between mean protein log concentration in the plasma isolated from whole human blood samples (*n* = 20) infected with the *Staphylococcus aureus* Newman strain precultured on Mueller–Hinton agar non-supplemented (A model—control); and supplemented with 1% (v/v) Tween 80 and *trans*-anethole at the subinhibitory concentration (5%, v/v, C model). The heatmap represents the r scores (+ 1 blue to − 0.5 white).

Figure 5 shows Pearson's correlation analysis between the analyzed mean log concentrations of proteins depending on the evaluated models (A—control and C). Only strong positive (r = + 0.8 or higher) or negative (r = -0.8 or lower) correlations with *P* < 0.05 values were considered. As shown, there was a strong positive correlation between the concentrations of CCL5 and CXCL1 (A model: r = 0.8364, *P* < 0.001; C model: r = 0.8729, *P* < 0.001) in both models. Furthermore, a strong positive correlation between TNF-α and CCL4 (r = 0.8509, *P* < 0.001) was additionally noticed in the C model.

## Discussion

*S. aureus,* during infection, provides the host with many PAMPs, including PG, lipoteichoic acid, or CpG DNA, which PRRs recognizes^19^. According to the literature, TLRs can recognize a wide range of PAMPs, including PG^20^. Our previous studies hypothesized that the observed changes in the surface structure (mainly in PG) of the TA-treated *S. aureus* Newman strain might affect TLR2 activation^17^. In that study, it has been noted, among other things, a high concentration of CXCL8 in plasma, whose release is mediated by a TLR2-dependent manner^21^. Later studies showed that TA-treated *S. aureus* Newman added to the blood samples of healthy participants caused upregulation of the *TLR2* gene in lymphocytes compared to blood samples infected with non-treated bacteria^18^. Nevertheless, in the current study, no significant changes in plasma TLR2 concentrations have been found after TA-treated *S. aureus* Newman human blood stimulation compared to control (non-treated bacteria). Interestingly, these bacteria led to a statistically significant increase in plasma levels of another PRR—PGLYRP1, for which, however, has been observed no correlation with the other proteins among A and C models. PGLYRP1 is a PG's recognizing membrane protein (it can also be secreted outside), which is an activator for the triggering receptor expressed on myeloid cells 1 (TREM-1) present on many immune cells, including, e.g., neutrophils, monocytes, NK cells, DCs, B and T cells^22,23^. According to the literature, TREM-1 activated by N3 peptide (a part of PGLYRP1) within the first hours influences the increased expression of *IL1B*, *IL6*, *TNF*, *IL2,* and *IFN* genes in human peripheral blood mononuclear cells^24^. Moreover, it has been shown that PGLYRP1 can take an active role in the intracellular killing of bacteria by macrophages^25^.

According to the literature, polymorphonuclear leukocytes (PMNs) are the primary source of MMPs, and *S. aureus*’ PG can affect the induction of this enzyme levels^4,26^. Because it has been revealed a strong response from neutrophils against the morphologically altered *S. aureus* Newman strain by TA in our previous studies^17^, in the current study, we decided to analyze the effect of TA-treated bacteria on MMP-8 (also known as neutrophil collagenase or collagenase-2) production. MMP-8 is a preformed protein located in specific granules in neutrophils, and its primary function is the degradation of types I–III collagens^27^. It is also worth mentioning that MMP-8 may play a pivotal role in wound healing and tissue reconstruction during inflammation. It was noted in a study by Gutiérrez-Fernández et al*.*^28^, in which MMP-8-deficient mice characterized delayed wound healing. Based on the results obtained in the current study, it has been observed that statistically increased production of MMP-8 in human blood samples stimulated with TA-treated bacteria compared to samples infected with non-treated bacteria. Generally, it has been noticed that adding *S. aureus* Newman to blood resulted in statistically increased production of MMP-8 compared to non-infected samples. Moreover, the present study did not report a correlation between MMP-8 and other proteins analyzed in the A and C models. Thirkettle et al*.*^29^ found that MMP-8 can affect innate immune mechanisms during inflammation, including activating chemokines (e.g., CXCL8), leading to increased neutrophil chemotaxis. In addition, other research proved that MMP-8 affects the increased production of IL-1β, TNF-α, and CD154 (CD40L)^30^.

In the current study, it was noted that blood samples isolated from healthy participants and then infected with TA-treated *S. aureus* Newman characterized in statistically increased expression of cytokines (IL-1β, IL-6, TNF-α, IL-12, G-CSF) and chemokines (C5a, CCL2—CCL4, CXCL1, CXCL2) in plasma compared to control. In addition, this model observed a strong positive correlation between CCL5 and CXCL1 and TNF-α and CCL4. Interestingly, Braunersreuther et al*.*^31^ demonstrated a direct role for CCL5 in CXCL1 synthesis, while Ahmad et al*.*^32^ showed that TNF-α increased CCL4 expression in human monocytes and macrophages. It was also revealed that the most abundantly expressed cytokine in all models was IL-12, mainly produced by macrophages, neutrophils, B cells, and DCs^33^. Literature data also indicate that it stimulates neutrophils to produce CXCL8^34^. In addition, the most abundantly expressed chemokine was C5a, formed by complement activation pathways in plasma or by cleavage of the C5 molecule by serine proteases derived, e.g., from neutrophils conducting the phagocytosis process^35^. Moreover, the activity of this process may also be determined by the presence of G-CSF^36^, for which statistically higher concentrations were observed in samples infected with TA-treated *S. aureus* Newman compared to the control.

On the contrary, IL-2, IFN-γ (secreted by Th1 cells), IL-17 (secreted by Th17 cells), and CCL1 (secreted by various immune cells, including T cells) were weakly expressed in all studied models^37,38^. It is also worth noting that in the present study, it was revealed that IL-1α concentration values were below the detection threshold in all models. According to the literature, IL-1α is produced by many cells, including neutrophils, keratinocytes, epithelial/endothelial cells, and lymphocytes^39^. The IL-1α results observed in this study may indicate that its activation may require a longer incubation time of blood with bacteria. In addition, it is worth highlighting here the aspect of the interaction between IL-17 and IFN-γ, which, according to the current literature cooperate with each other and may play a pivotal role in host protection against *S. aureus*^40^. In the current study, TA-treated *S. aureus* showed that the expression of these two cytokines in both control and treatment models was very insignificant. Nevertheless, as with IL-1α, incubation time is very important here. Usually, a strong correlation between these cytokines is observed after prolonged exposure to bacteria. For example, studies performed by Cheng et al.^41^ and Bartsch et al.^42^ showed a significant increase in IL-17 and IFN-γ within 6 h and 3 days after infection with *S. aureus*, respectively. Hence, further studies are required to demonstrate the possible correlation of individual cytokines over time.

As mentioned above, in our previous study, it has been noted that TA-treated *S. aureus* Newman added to human blood increased the efficiency of selected innate immune mechanisms^17^. Importantly, it has also been found changes in the surface structure (mainly in PG) of TA-treated *S. aureus*. Thus, we assumed in the current study that these changes could induce IL-1β, IL-6, TNF-α, IL-12, G-CSF, C5a, CCL2—CCL4, CXCL1, CXCL2 production in PGLYRP-1-dependent manner which through TREM-1 activation, could lead to the induction of pro-inflammatory cytokine release, initiating a cascade of immune network. Nevertheless, further research is required to confirm this. The role of MMP-8, the concentration of which can increase after activation by PG and which can also be an initiator of immune activation, cannot be overlooked here either^4,26^. In general, PMNs (including mainly neutrophils) are the first line of defense against *S. aureus*. Neutrophils respond to various proteins within minutes of contact with these bacteria, including chemokines and cytokines. These proteins are used by immune cells to communicate and organize the immune response. As reported in the literature, properties that enhance phagocyte activity, including neutrophil recruitment, intracellular killing, and, interestingly, the formation of NETs, have been demonstrated for the proteins mentioned above^29,36,43–47^.

Because the present study noted an increased immunomodulatory activity of the immune system in response to TA-treated *S. aureus*, other studies reporting a similar effect are worth mentioning. Nevertheless, the available literature lacks studies conducted by other researchers on the direct pre-treatment of bacteria (or their components) with EOs/EOCs and then stimulating the immune system with these modified antigens. Nonetheless, few studies have been reported describing the upregulation of cytokines and chemokines produced by immune cells after co-stimulation with non-treated antigens and EOs/EOCs. For instance, a study performed by Miastkowska et al*.*^48^ showed that co-stimulation with lavender EO and LPS resulted in increased IL-1β, IL-6, and IL-8 in the HaCaT cell line in comparison to LPS-stimulated cells. Another study reported an increase in IL-10 concentration produced by mouse macrophages stimulated with LPS and clove (50 µg) as well as LPS and lemongrass (100 µg) compared to a sample stimulated with LPS alone^49,50^. A study performed by Liu et al*.*^51^ showed that garlic extract (at various concentrations) caused increased LPS-induced production of IL-1β, TNF-α, and TGF-β by alveolar macrophages isolated from healthy donor pigs. Interestingly, the authors also noted an increase in IL-1β and TGF-β concentration after macrophage stimulation with LPS and anethole (at 25 and 100 µg/ml).

Although experiments involving live microorganisms and active substances in whole-blood models best imitate what happens during an inflammatory reaction induced by pathogens, unfortunately, it is complicated to analyze the mechanism of immune cells' response accurately. There are also questions about the production of virulence factors (including factors responsible for evasion mechanisms), the rate of activation of immune cells, and the general condition of the immune system^52,53^. Therefore, in our proposed model of the effect of TA-treated *S. aureus* Newman on the production of the proteins mentioned above, it can be concluded that it stimulates the immune response. Nevertheless, further in-depth studies need to be provided to reveal the underlying molecular mechanisms for the immunomodulatory effects of TA-treated bacteria in more detail.

Our next step will be to analyze the immune response against TA-treated *S. aureus* over time. In the present study, we found that a cascade of selected proteins released during the initial phase (within 2 h) of the inflammatory response causes activation of the immune system. However, we do not know whether this activation will be regulated and cease promptly after the destruction of the bacteria or whether it may lead to uncontrolled cytokine shedding. Therefore, we need to consider many aspects, including the effect of TA on the expression of bacterial toxins (e.g., superantigens) of *S. aureus* or the effect of TA-treated *S. aureus* on the viability of immune cells, which may be necessary for the process of pyroptosis. To date, we have shown that incubation (within 2 h) of TA-treated bacteria with blood did not affect lymphocyte viability^18^. However, these results are encouraging for further analyses, which are currently being performed in our laboratory.

## Conclusions

In conclusion, in the current study, we proved the immediate (within the first 2 h) immunomodulatory effect of TA-treated *S. aureus* Newman on increasing IL-1β, IL-6, TNF-α, IL-12, G-CSF, C5a, CCL2, CCL3, CCL4, CXCL1, CXCL2, MMP-8 and PGLYRP1 levels in plasma. Moreover, we demonstrated an association between TNF-α and CCL4 in a blood model infected with TA-treated cells. Nevertheless, more research is warranted to find underlying mechanisms involved in the effects of TA-treated *S. aureus* Newman in human blood, mainly whether the observed “immunity boost” can be regulated after bacteria elimination. Therefore, the potential of TA should be further explored to understand under which conditions it might be a valuable tool for treating or preventing infections caused by *S. aureus*.

## Supplementary Information

Supplementary Information.

## Data Availability

The raw data supporting the conclusions of this article will be made available by the corresponding author (P.K.) without undue reservation.
