# Supplementary material for: Immunomodulatory effects of trans-anethole-treated Staphylococcus aureus Newman strain
Source: Sci Rep. 2023 Apr 27;13:6881. doi: 10.1038/s41598-023-34138-3 (PMC10140024; doi:10.1038/s41598-023-34138-3)
Supplement: Supplementary file 1 — Supplementary Information. [file 41598_2023_34138_MOESM1_ESM.docx]

**Table S1.** Characteristics of participants (*n* = 20) by age, sex, and hematological parameters.

| **Parameter** | **Participant no.** | | | | | | | | | | | | | | | | | | | | **RI** | |
| --- | --- | --- | --- | --- | --- | --- | --- | --- | --- | --- | --- | --- | --- | --- | --- | --- | --- | --- | --- | --- | --- | --- |
|  | **1.** | **2.** | **3.** | **4.** | **5.** | **6.** | **7.** | **8.** | **9.** | **10.** | **11.** | **12.** | **13.** | **14.** | **15.** | **16.** | **17.** | **18.** | **19.** | **20.** |  |  |
| Gender | F | F | M | F | F | F | F | F | F | F | F | F | F | F | F | F | F | F | F | M |  |  |
| Age | 42 | 44 | 31 | 21 | 36 | 64 | 61 | 27 | 56 | 25 | 57 | 45 | 46 | 45 | 34 | 30 | 61 | 53 | 30 | 27 |  |  |
| WBC (g/l) | 5.65 | 7.23 | 7.23 | 4.53 | 7.68 | 8.93 | 4.81 | 5.13 | 5.27 | 7.09 | 5.09 | 7.19 | 7.68 | 7.26 | 5.09 | 7.75 | 4.60 | 6.44 | 8.14 | 6.08 | 4.00 — 10.00 | |
| LYM (g/l) | 1.58 | 1.64 | 2.63 | 1.70 | 2.04 | 3.36 | 1.71 | 2.95 | 2.12 | 2.85 | 1.08 | 2.98 | 2.55 | 2.48 | 1.76 | 2.52 | 1.38 | 2.27 | 0.83 | 2.10 | 0.80 — 4.00 | |
| LYM (%) | 28.0 | 22.7 | 36.4 | 37.5 | 26.6 | 37.6 | 35.6 | 37.5 | 40.2 | 40.2 | 21.2 | 41.4 | 33.2 | 34.2 | 34.6 | 32.5 | 38.3 | 35.2 | 20.2 | 34.5 | 20.0 — 45.0 | |
| NEU (g/l) | 3.48 | 5.02 | 3.72 | 2.37 | 4.58 | 4.74 | 2.37 | 2.77 | 2.34 | 3.63 | 3.38 | 3.63 | 4.53 | 3.71 | 2.64 | 4.36 | 2.75 | 3.47 | 6.49 | 3.35 | 2.00 — 6.90 | |
| NEU (%) | 61.6 | 69.4 | 51.4 | 52.4 | 59.7 | 53.1 | 49.3 | 37.5 | 44.3 | 51.2 | 66.4 | 50.6 | 59.0 | 51.1 | 51.8 | 56.3 | 48.6 | 54.0 | 69.8 | 55.1 | 37.0 — 70.0 ^F^ | 40.0 — 70.0 ^M^ |
| MON (g/l) | 0.44 | 0.47 | 0.54 | 0.31 | 0.81 | 0.70 | 0.70 | 0.31 | 0.43 | 0.42 | 0.46 | 0.29 | 0.44 | 0.86 | 0.42 | 0.67 | 0.29 | 0.53 | 0.75 | 0.51 | 0.10 — 0.90 | |
| MON (%) | 7.80 | 6.50 | 7.50 | 6.80 | 8.50 | 7.80 | 2.31 | 6.00 | 8.20 | 5.90 | 9.00 | 4.00 | 5.70 | 8.80 | 8.30 | 8.60 | 8.10 | 8.20 | 9.00 | 8.40 | 2.00 — 9.00 | |
| EOS (g/l) | 0.11 | 0.06 | 0.28 | 0.11 | 0.21 | 0.07 | 0.40 | 0.06 | 0.34 | 0.10 | 0.13 | 0.24 | 0.12 | 0.14 | 0.21 | 0.16 | 0.15 | 0.11 | 0.05 | 0.09 | 0.00 — 0.70 | |
| EOS (%) | 1.90 | 0.80 | 3.90 | 2.40 | 2.70 | 0.80 | 0.05 | 1.20 | 6.50 | 1.40 | 2.60 | 3.30 | 1.60 | 1.90 | 4.10 | 2.10 | 4.20 | 1.70 | 0.60 | 1.50 | 0.00 — 7.00 | |
| BAS (g/l) | 0.04 | 0.04 | 0.06 | 0.04 | 0.04 | 0.06 | 0.05 | 0.04 | 0.04 | 0.09 | 0.04 | 0.05 | 0.04 | 0.07 | 0.06 | 0.04 | 0.03 | 0.06 | 0.02 | 0.03 | 0.00 — 0.20 | |
| BAS (%) | 0.70 | 0.60 | 0.80 | 0.90 | 0.50 | 0.70 | 1.00 | 0.80 | 0.80 | 1.30 | 0.80 | 0.70 | 0.50 | 1.00 | 1.20 | 0.50 | 0.80 | 0.90 | 0.20 | 0.50 | 0.00 — 1.50 | |
| IG (g/l) | 0.01 | 0.02 | 0.03 | 0.01 | 0.02 | 0.01 | 0.01 | 0.01 | 0.02 | 0.01 | 0.02 | 0.02 | 0.02 | 0.02 | 0.01 | 0.02 | 0.00 | 0.02 | 0.02 | 0.02 | 0.00 — 0.03 | |
| IG (%) | 0.20 | 0.30 | 0.50 | 0.20 | 0.30 | 0.10 | 0.20 | 0.20 | 0.40 | 0.10 | 0.40 | 0.30 | 0.30 | 0.30 | 0.20 | 0.30 | 0.00 | 0.30 | 0.20 | 0.30 | 0.00 — 0.50 | |
| RBC (T/l) | 4.70 | 4.96 | 5.29 | 4.34 | 4.57 | 4.49 | 4.09 | 4.33 | 4.17 | 4.59 | 4.23 | 4.34 | 4.27 | 4.55 | 4.37 | 4.52 | 4.54 | 4.06 | 4.43 | 5.41 | 4.00 — 5.00 ^F^ | 4.50 — 5.50 ^M^ |
| HGB (mmol/l) | 8.3 | 9.2 | 9.4 | 7.8 | 8.6 | 8.4 | 7.9 | 8.2 | 8.5 | 8.4 | 8.8 | 7.9 | 8.5 | 8.7 | 7.8 | 8.3 | 8.2 | 7.7 | 8.5 | 10.7 | 7.7 — 10.0 ^F^ | 8.4 — 11.0 ^M^ |
| HCT (l/l) | 0.39 | 0.44 | 0.47 | 0.37 | 0.41 | 0.40 | 0.39 | 0.39 | 0.40 | 0.37 | 0.41 | 0.37 | 0.40 | 0.43 | 0.38 | 0.38 | 0.40 | 0.37 | 0.40 | 0.47 | 0.37 — 0.47 ^F^ | 0.40 — 0.54 ^M^ |
| MCV (fl) | 83.4 | 87.9 | 88.5 | 84.8 | 89.1 | 89.1 | 94.1 | 89.1 | 95.4 | 86.7 | 97.2 | 84.6 | 93.2 | 93.6 | 86.3 | 84.7 | 87.0 | 88.4 | 90.1 | 83.4 | 81.0 — 101.0 ^F^ | 80.0 — 94.0 ^M^ |
| MCH (fmol) | 1.77 | 1.86 | 1.78 | 1.80 | 1.88 | 1.87 | 1.93 | 1.89 | 2.04 | 1.83 | 2.08 | 1.82 | 1.99 | 1.91 | 1.79 | 1.84 | 1.81 | 1.82 | 1.92 | 1.91 | 1.70 — 2.10 | |
| MCHC (mmol/l) | 21.2 | 21.1 | 20.1 | 21.2 | 21.1 | 21.0 | 20.5 | 21.2 | 21.4 | 21.1 | 21.4 | 21.5 | 21.4 | 20.4 | 20.7 | 21.7 | 20.8 | 20.6 | 21.3 | 22.9 | 19.0 — 23.0 | |
| RDW (%) | 12.9 | 12.2 | 11.8 | 12.7 | 11.5 | 13.2 | 13.1 | 12.1 | 12.1 | 11.7 | 13.2 | 13.7 | 13.1 | 12.6 | 12.9 | 12.6 | 12.4 | 12.7 | 12.6 | 12.1 | 11.5 — 14.5 | |
| PLT (g/l) | 195 | 376 | 288 | 209 | 254 | 291 | 279 | 261 | 225 | 263 | 288 | 355 | 295 | 253 | 215 | 294 | 155 | 208 | 172 | 273 | 150 — 400 | |
| MPV (fl) | 11.8 | 10.2 | 10.8 | 12.2 | 11.7 | 10.0 | 9.4 | 10.8 | 10.8 | 10.8 | 10.0 | 10.7 | 9.8 | 12.0 | 9.8 | 9.4 | 11.2 | 10.2 | 11.4 | 10.1 | 9.4 — 12.6 | |

F — female; M — male; WBC — white blood cells; LYM — lymphocytes; NEU — neutrophils; MON — monocytes; EOS — eosinophils; BAS — basophils; IG — immature granulocytes; RBC — red blood cells; HGB — hemoglobin; HCT — hematocrit; MCV — mean corpuscular volume; MCH — mean corpuscular hemoglobin; MCHC — mean corpuscular hemoglobin concentration; RDW — red blood cell distribution width; PLT — platelets; MPV — mean platelet volume; RI — reference interval.

**Table S2.** Statistical parameters of cytokine, chemokine, and other protein concentrations in the plasma isolated from whole human blood samples (*n* = 20) non-infected (N model) and infected with the *Staphylococcus aureus* Newman strain precultured on Mueller-Hinton agar: non-supplemented (A model – control); supplemented with 1% (v/v) Tween 80 (B model); supplemented with 1% (v/v) Tween 80 and *trans*-anethole at the subinhibitory concentration (5%, v/v, C model).

| **Statistical**  **parameters** | **Model** | **Proteins** | | | | | | | | | | | | | | | | | | | | | |
| --- | --- | --- | --- | --- | --- | --- | --- | --- | --- | --- | --- | --- | --- | --- | --- | --- | --- | --- | --- | --- | --- | --- | --- |
|  |  | **Cytokines** | | | | | | | | **Chemokines** | | | | | | | | | | | **Other** | | |
|  |  | **IL-1β** | **IL-2** | **IL-6** | **IL-12** | **IL-17** | **TNF-α** | **IFN-γ** | **G-CSF** | **C5a** | **CCL1** | **CCL2** | **CCL3** | **CCL4** | **CCL5** | **CXCL1** | **CXCL2** | **CXCL9** | **CXCL10** | **CXCL11** | **MMP-8** | **TLR2** | **PGLYRP1** |
| Mean | N | 53.7 | 0.8 | 16.1 | 559.5 | 15.8 | 4.0 | 1.9 | 115.1 | 6096.5 | 4.7 | 443.5 | 784.4 | 887.4 | 1596.3 | 627.4 | 162.7 | 1129.1 | 140.1 | 69.3 | 349.4 | 286.9 | 252.7 |
|  | A | 50.6 | 0.9 | 6.5 | 615.2 | 17.2 | 48.3 | 1.9 | 94.2 | 5911.8 | 4.6 | 422.5 | 616.2 | 772.2 | 1166.9 | 556.6 | 170.7 | 1507.1 | 127.3 | 75.0 | 4339.4 | 297.6 | 344.1 |
|  | B | 49.0 | 0.8 | 11.2 | 726.6 | 13.0 | 82.2 | 1.7 | 105.3 | 5981.5 | 4.7 | 518.3 | 841.6 | 1496.1 | 1627.9 | 673.0 | 364.8 | 1379.0 | 138.4 | 92.8 | 3560.5 | 303.8 | 333.6 |
|  | C | 79.1 | 0.9 | 41.0 | 1647.1 | 16.5 | 367.6 | 2.2 | 183.4 | 9014.3 | 6.1 | 600.7 | 1255.7 | 2830.8 | 1520.8 | 900.4 | 574.8 | 1999.0 | 152.3 | 108.5 | 9168.8 | 325.9 | 544.8 |
| SD | N | 27.9 | 0.5 | 19.9 | 336.2 | 6.8 | 3.2 | 2.2 | 89.1 | 2940.8 | 2.3 | 203.3 | 526.0 | 1083.3 | 1690.0 | 402.2 | 182.5 | 1648.9 | 68.0 | 64.2 | 221.5 | 142.1 | 64.4 |
|  | A | 19.2 | 0.7 | 9.3 | 476.9 | 7.1 | 47.5 | 2.3 | 44.0 | 2912.3 | 3.5 | 210.0 | 217.5 | 432.7 | 1263.9 | 485.7 | 148.1 | 1697.2 | 76.1 | 67.5 | 3149.0 | 152.3 | 78.2 |
|  | B | 28.9 | 0.6 | 13.1 | 660.8 | 5.1 | 39.7 | 1.9 | 65.0 | 3303.5 | 3.0 | 195.2 | 344.0 | 848.0 | 1717.0 | 378.8 | 263.3 | 1631.1 | 68.7 | 75.8 | 2529.5 | 169.4 | 72.0 |
|  | C | 31.5 | 0.7 | 22.5 | 1092.8 | 7.0 | 189.9 | 2.4 | 63.5 | 3756.9 | 2.9 | 198.2 | 501.3 | 1603.3 | 1569.0 | 320.1 | 365.5 | 1621.3 | 65.8 | 79.4 | 3746.4 | 181.5 | 105.8 |
| Median | N | 34.5 | 0.6 | 2.4 | 319.0 | 10.9 | 3.0 | 0.8 | 95.3 | 6455.0 | 4.7 | 435.0 | 492.0 | 344.0 | 201.8 | 458.0 | 71.6 | 340.1 | 142.7 | 38.2 | 291.8 | 213.1 | 252.3 |
|  | A | 34.5 | 0.6 | 2.4 | 319.0 | 10.9 | 25.8 | 0.9 | 95.3 | 5560.0 | 3.9 | 386.0 | 544.0 | 584.0 | 473.2 | 274.0 | 149.2 | 902.0 | 112.3 | 43.6 | 3623.1 | 214.9 | 338.1 |
|  | B | 34.5 | 0.6 | 2.4 | 319.0 | 10.9 | 85.9 | 0.8 | 95.3 | 6045.0 | 4.4 | 569.0 | 789.0 | 1409.0 | 750.7 | 595.0 | 246.0 | 767.2 | 136.3 | 87.6 | 3026.0 | 213.5 | 325.0 |
|  | C | 81.3 | 0.7 | 40.0 | 1006.0 | 10.9 | 310.0 | 1.0 | 170.0 | 10030.0 | 5.2 | 615.0 | 1158.0 | 2674.0 | 762.6 | 949.0 | 410.0 | 1429.4 | 142.0 | 107.6 | 8710.7 | 226.3 | 567.0 |
| IQR | N | 39.6 | 0.4 | 33.2 | 687.0 | 13.9 | 0.0 | 2.6 | 120.1 | 5397.5 | 3.0 | 241.0 | 623.0 | 611.5 | 2832.5 | 851.5 | 286.7 | 996.8 | 58.6 | 106.3 | 181.5 | 163.5 | 67.1 |
|  | A | 32.5 | 0.5 | 0.0 | 687.0 | 13.9 | 45.5 | 2.1 | 70.1 | 4872.5 | 4.6 | 298.0 | 295.0 | 762.0 | 1610.9 | 946.8 | 79.4 | 1500.6 | 69.7 | 116.4 | 3621.2 | 176.8 | 108.5 |
|  | B | 32.5 | 0.4 | 12.3 | 687.0 | 0.0 | 44.3 | 2.6 | 84.2 | 5731.3 | 4.5 | 218.5 | 399.5 | 1172.0 | 2938.3 | 824.0 | 316.5 | 1237.2 | 51.8 | 138.1 | 2556.9 | 128.7 | 105.8 |
|  | C | 28.5 | 0.8 | 37.8 | 1706.0 | 13.9 | 350.0 | 2.2 | 32.0 | 4736.3 | 2.7 | 215.5 | 754.5 | 3078.5 | 2424.8 | 637.0 | 613.0 | 1738.0 | 43.9 | 117.9 | 5801.6 | 233.1 | 198.5 |
| CV (%) | N | 52.0 | 69.8 | 123.8 | 60.1 | 43.1 | 79.5 | 115.4 | 77.4 | 48.2 | 49.7 | 45.9 | 67.1 | 122.1 | 105.9 | 64.1 | 112.2 | 146.0 | 48.5 | 92.7 | 63.4 | 49.5 | 25.5 |
|  | A | 38.0 | 77.7 | 143.1 | 77.5 | 41.4 | 98.3 | 118.5 | 46.7 | 49.3 | 76.4 | 49.7 | 35.3 | 56.0 | 108.3 | 87.3 | 86.8 | 112.6 | 59.8 | 89.9 | 72.6 | 51.2 | 22.7 |
|  | B | 59.0 | 72.8 | 117.2 | 91.0 | 39.2 | 48.3 | 109.1 | 61.7 | 55.2 | 62.6 | 37.7 | 40.9 | 56.7 | 105.5 | 56.3 | 72.2 | 118.3 | 49.7 | 81.6 | 71.0 | 55.8 | 21.6 |
|  | C | 39.8 | 78.1 | 55.0 | 66.3 | 42.4 | 51.7 | 107.6 | 34.6 | 41.7 | 47.6 | 33.0 | 39.9 | 56.6 | 103.2 | 35.6 | 63.6 | 81.1 | 43.2 | 73.2 | 40.9 | 55.7 | 19.4 |
| Min | N | 15.3 | 0.3 | 2.4 | 319.0 | 10.9 | 3.0 | 0.2 | 0.9 | 1315.0 | 1.7 | 123.3 | 440.0 | 37.1 | 163.4 | 274.0 | 9.7 | 135.6 | 60.2 | 18.0 | 104.2 | 130.2 | 143.1 |
|  | A | 34.5 | 0.3 | 2.4 | 319.0 | 10.9 | 3.0 | 0.2 | 49.9 | 1360.0 | 1.7 | 123.3 | 164.8 | 252.0 | 195.5 | 25.1 | 9.7 | 223.4 | 36.7 | 18.0 | 646.1 | 187.4 | 229.3 |
|  | B | 5.2 | 0.3 | 2.4 | 319.0 | 10.9 | 4.0 | 0.2 | 0.9 | 1330.0 | 1.7 | 145.9 | 440.0 | 430.0 | 189.2 | 101.2 | 9.7 | 180.1 | 52.1 | 18.0 | 881.0 | 173.0 | 214.3 |
|  | C | 34.5 | 0.3 | 14.7 | 319.0 | 10.9 | 86.6 | 0.2 | 95.3 | 1460.0 | 1.7 | 198.7 | 716.0 | 724.0 | 176.4 | 388.0 | 149.2 | 475.6 | 68.4 | 18.0 | 2164.5 | 191.7 | 358.1 |
| Max | N | 95.5 | 2.2 | 52.5 | 1006.0 | 24.8 | 15.8 | 7.8 | 260.0 | 9880.0 | 8.4 | 780.0 | 1972.0 | 3110.0 | 4868.7 | 1224.0 | 518.0 | 6661.4 | 332.0 | 220.0 | 941.5 | 606.3 | 384.0 |
|  | A | 95.5 | 2.9 | 35.6 | 2120.0 | 24.8 | 152.3 | 7.3 | 202.0 | 10510.0 | 14.7 | 816.0 | 1208.0 | 1574.0 | 4178.7 | 1188.0 | 704.0 | 7024.7 | 340.0 | 220.0 | 12071.2 | 736.8 | 488.7 |
|  | B | 95.5 | 2.4 | 52.5 | 2120.0 | 24.8 | 146.0 | 5.8 | 232.0 | 10705.0 | 13.4 | 782.0 | 1762.0 | 3700.0 | 4908.1 | 1154.0 | 918.0 | 6675.7 | 332.0 | 266.0 | 9229.2 | 709.7 | 466.7 |
|  | C | 156.7 | 3.1 | 92.5 | 3472.0 | 24.8 | 598.0 | 8.0 | 362.0 | 15395.0 | 16.0 | 938.0 | 2250.0 | 4798.0 | 4756.3 | 1256.0 | 1302.0 | 7000.0 | 336.0 | 324.0 | 15402.4 | 801.2 | 676.7 |

SD — standard deviation; IQR — interquartile range; CV — coefficient of variation; Min — minimum value; Max — maximum value.

**Table S3.** *P*-values of the model data obtained in the experiment.

| **Proteins** | ***P*-values of the comparison models** | | | | | |
| --- | --- | --- | --- | --- | --- | --- |
|  | **N *vs*. A** | **N *vs.* B** | **N *vs.* C** | **A *vs.* B** | **A *vs.* C** | **B *vs.* C** |
| **Cytokines** | | | | | | |
| IL-1β | >0.9999 | >0.9999 | 0.0514 | >0.9999 | 0.0243 | 0.0158 |
| IL-2 | >0.9999 | >0.9999 | >0.9999 | >0.9999 | >0.9999 | >0.9999 |
| IL-6 | >0.9999 | >0.9999 | 0.0005 | >0.9999 | <0.0001 | 0.0002 |
| IL-12 | >0.9999 | >0.9999 | 0.0032 | >0.9999 | 0.0027 | 0.0063 |
| IL-17 | >0.9999 | >0.9999 | >0.9999 | 0.2771 | >0.9999 | 0.5798 |
| TNF-α | 0.0066 | <0.0001 | <0.0001 | >0.9999 | <0.0001 | 0.0078 |
| IFN-γ | >0.9999 | >0.9999 | >0.9999 | >0.9999 | >0.9999 | >0.9999 |
| G-CSF | >0.9999 | >0.9999 | 0.0207 | >0.9999 | 0.0013 | 0.0073 |
| **Chemokines** | | | | | | |
| C5a | >0.9999 | >0.9999 | 0.0454 | >0.9999 | 0.0310 | 0.0386 |
| CCL1 | >0.9999 | >0.9999 | 0.8421 | >0.9999 | 0.3452 | 0.5558 |
| CCL2 | >0.9999 | >0.9999 | 0.1228 | 0.5928 | 0.0396 | >0.9999 |
| CCL3 | >0.9999 | 0.6422 | 0.0005 | 0.3342 | 0.0001 | 0.1140 |
| CCL4 | >0.9999 | 0.0099 | <0.0001 | 0.1422 | 0.0002 | 0.3494 |
| CCL5 | >0.9999 | >0.9999 | >0.9999 | >0.9999 | >0.9999 | >0.9999 |
| CXCL1 | >0.9999 | >0.9999 | 0.0921 | >0.9999 | 0.0035 | 0.1227 |
| CXCL2 | >0.9999 | 0.0225 | <0.0001 | 0.0733 | 0.0001 | 0.4542 |
| CXCL9 | 0.4511 | >0.9999 | 0.0055 | >0.9999 | 0.7496 | 0.2335 |
| CXCL10 | >0.9999 | >0.9999 | >0.9999 | >0.9999 | 0.9358 | >0.9999 |
| CXCL11 | >0.9999 | >0.9999 | 0.5269 | >0.9999 | 0.8855 | >0.9999 |
| **Other** | | | | | | |
| MMP-8 | <0.0001 | 0.0002 | <0.0001 | >0.9999 | 0.0450 | 0.0081 |
| TLR2 | >0.9999 | >0.9999 | >0.9999 | >0.9999 | >0.9999 | >0.9999 |
| PGLYRP1 | 0.2148 | 0.4218 | <0.0001 | >0.9999 | 0.0089 | 0.0032 |

The plasma samples (*n* = 20) non-infected (model N) and infected with the *Staphylococcus aureus* Newman strain precultured on Mueller-Hinton agar: non-supplemented (A model – control); supplemented with 1% (v/v) Tween 80 (B model); supplemented with 1% (v/v) Tween 80 and *trans*-anethole at the subinhibitory concentration (5%, v/v, C model).
